# Nucleocapsid and Spike Proteins of the Coronavirus SARS-CoV-2 Induce *IL6* in Monocytes and Macrophages—Potential Implications for Cytokine Storm Syndrome

**DOI:** 10.3390/vaccines9010054

**Published:** 2021-01-15

**Authors:** Iwona Karwaciak, Anna Sałkowska, Kaja Karaś, Jarosław Dastych, Marcin Ratajewski

**Affiliations:** 1 Laboratory of Transcriptional Regulation, Institute of Medical Biology, Polish Academy of Sciences, 93-232 Lodz, Poland; isachrajda@cbm.pan.pl; 2 Laboratory of Epigenetics, Institute of Medical Biology, Polish Academy of Sciences, 93-232 Lodz, Poland; asalkowska@cbm.pan.pl (A.S.); kkaras@cbm.pan.pl (K.K.); 3 Laboratory of Cellular Immunology, Institute of Medical Biology, Polish Academy of Sciences, 93-232 Lodz, Poland; jdastych@cbm.pan.pl

**Keywords:** SARS-COV-2, COVID-19, monocytes, macrophages, IL-6, cytokine storm

## Abstract

The pandemic of the new coronavirus SARS-CoV-2 (severe acute respiratory syndrome coronavirus 2) has led to the deaths of more than 1.5 million people worldwide. SARS-CoV-2 causes COVID-19, which exhibits wide variation in the course of disease in different people, ranging from asymptomatic and mild courses to very severe courses that can result in respiratory failure and death. Despite the rapid progression of knowledge, we still do not know how individual cells of the immune system interact with the virus or its components, or how immune homeostasis becomes disrupted, leading to the rapid deterioration of a patient’s condition. In the present work, we show that SARS-CoV-2 proteins induce the expression and secretion of IL-6 by human monocytes and macrophages, the first line cells of antiviral immune responses. IL-6 may play a negative role in the course of COVID-19 by inhibiting Th1-dependent immunity and stimulating Th17 lymphocytes, thus leading to an increased probability of a cytokine storm.

## 1. Introduction

A severe acute respiratory syndrome coronavirus 2 (SARS-CoV-2) virus similar to other severe acute respiratory syndrome coronaviruses (SARS-CoV-1 and MERS-CoV) has caused outbreaks around the world [1,2], but the scale of these outbreaks is incomparable to that of previous outbreaks, leading the virus to become a major problem in global healthcare. SARS-CoV-2 causes a disease called COVID-19 (coronavirus disease 2019) [3]. Most patients have a mild disease course and good prognosis [4], with typical symptoms including fever, dry unproductive cough, fatigue, and dyspnea [5]; however, other patients may develop pneumonia, leading to acute respiratory or multiorgan failure and death [6]. Until now, it has remained unclear why different people exhibit a wide variety of symptoms and course of disease, although age and underlying diseases might increase the probability of a severe COVID-19 course [6]. Despite many attempts and very rapid progress, our knowledge about the immune response to SARS-CoV-2 remains incomplete, and understanding the mechanisms of COVID-19 pathogenesis is crucial for revealing new possibilities of successfully treating this disease.

One of the most dangerous consequences of coronavirus SARS-CoV-2 infection (like other viruses, e.g., influenza [7]) is a cytokine storm caused by immune imbalance and self-perpetuating inflammatory reactions [8] that leads to acute respiratory distress syndrome (ARDS), which is the main cause of death of patients infected with SARS-CoV-2, SARS-CoV-1, and MERS-CoV [9,10,11]. Numerous cytokines and chemokines have been associated with the ARDS caused by coronavirus infections [12,13,14,15,16]. Among these cytokines, IL-6 is particularly important in the persistence of the proinflammatory milieu [12,16]. Monocytes, which are one of the main sources of this cytokine at inflammatory sites [17], are found in large numbers in the bloodstream and are often the first cells of the immune system to come into contact with virus particles [18]. Furthermore, viral proteins derived from SARS-CoV-1 have been shown to upregulate proinflammatory cytokines in peripheral blood monocytes [19]. The exact mechanism responsible for the hyperactivation of monocytes and macrophages in COVID-19 is still unknown. However, it is possible that high levels of virus replication and delayed activation of INF-I (type I interferon) signaling leads to the accumulation of tissue infiltrating monocytes and macrophages, given that a reduced response from T lymphocytes triggers an uncontrolled cytokine release and cytokine storm inducing, for example, inflammatory injury and organ failure [20,21,22,23,24]. Interestingly, macrophages infected with SARS-CoV-2 and isolated postmortem from lymph nodes and spleens of COVID-19 patients have shown high levels of IL-6 expression [25]. These observations prompted us to investigate the effects of SARS-CoV-2 viral proteins on the expression of proinflammatory cytokines, particularly IL-6, in monocytes and monocyte-derived macrophages isolated from the peripheral blood of healthy unexposed donors. We found that nucleocapsid (N) protein and, to a lesser extent, spike (S) protein induce the expression of *IL6* and other proinflammatory cytokines, suggesting that these cells may be the initial source of this cytokine and participate in the development of cytokine storms during COVID-19 infection. This finding also suggests that inhibition of SARS-CoV-2-mediated IL-6 expression in monocytes and macrophages could be a target for novel treatment strategies for severe COVID-19 patients.

## 2. Materials and Methods 

### 2.1. Monocyte Isolation and Macrophage Differentiation 

Peripheral blood mononuclear cells (PBMCs) were isolated from buffy coats by centrifugation on a Ficoll density gradient. Blood was purchased from the Regional Center for Blood Donation and Blood Treatment, Łódź, Poland, and had been obtained from anonymous healthy donors. The plasma samples corresponding to the buffy coats were tested for the presence of anti-SARS-CoV2 antibodies using the Accu-Tell COVID-19 in vitro diagnostic IgG/IgM test (AccuBio Tech, Beijing, China) and the EDI^TM^ Novel Coronavirus COVID-19 IgG ELISA Kit (Epitope Diagnostics Inc., San Diego, CA, USA). All the serum samples were negative; thus, the isolated cells were treated as having been obtained from unexposed individuals. Monocytes were isolated using the Classical Monocyte Isolation Kit, human 130-117-337 from Miltenyi Biotec (Bergisch Gladbach, Germany). The cells were cultured in RPMI 1640 medium containing 10% FBS (fetal bovine serum) and 10% human AB serum (PAN Biotech, Aidenbach, Germany) for 3 days. For macrophage differentiation, monocytes were isolated as described above and then cultured in RPMI 1640 medium containing 10% FBS and 10% human AB serum (PAN Biotech) supplemented with 10 ng/mL GM-CSF (R&D Systems, Bio-Techne, Minneapolis, MN, USA) for 5 days.

### 2.2. SARS-CoV-2 Proteins

SARS-CoV-2 coronavirus proteins, COVID-19 Nucleocapsid Protein 32-190001 and recombinant 2019-nCoV S1 Protein (Active) 32-190005, were purchased from Abeomics (San Diego, CA, USA). Proteins were resuspended in phosphate-buffered saline (PBS), pH 7.4.

### 2.3. Real-Time RT-PCR

TRI Reagent (Molecular Research Center, Cincinnati, OH, USA) was used to extract RNA from the cells. The Maxima First Strand cDNA Synthesis Kit for RT-quantitative PCR (Thermo Fisher Scientific, Waltham, MA, USA) was used to reverse-transcribe 1 μg of RNA. The resulting cDNA were used as temple in real-time RT-PCR, which was run using SYBR Green I Master Mix on a LightCycler 480 (Roche, Basel, Switzerland). The cycling conditions were as follows: 95 °C for 5 min, followed by 55 cycles of 95 °C for 10 s, 60 °C for 10 s, and 72 °C for 20 s. The primer sequences used in this study were as follows: *IL6*, 5′-CCTGAACCTTCCAAAGATGG-3′ (forward) and 5′-GGTCAGGGGTGGTTATTGC-3′ (reverse), as previously described [26]; *IL1B*, 5′-GGACAGGATATGGAGCAACAAGTG-3′ (forward), and 5′- ACACGCAGGACAGGTACAGATTC-3′ (reverse); *IL10*, 5′- TTGCTGGAGGACTTTAAGGG-3′ (forward), and 5′-GGGAAGAAATCGATGACAGC-3′ (reverse); *TNF*, 5′-CCAGGCAGTCAGATCATCTTCTCG-3′ (forward), and 5′-ATCTCTCAGCTCCACGCCATTG-’3 (reverse), as previously described [27]; *RELA*, 5′-TATCAGTCAGCGCATCCAGACC-3′ (forward), and 5′-CGCTGCTCTTCTATAGGAACTTGG-3′ (reverse); *NFKB1*, 5′- CCTCCACAAGGCAGCAAATAGACG-3′ (forward), and 5′-AGCTGAGTTTGCGGAAGGATGTC-3′ (reverse) as previously described [28]; *NFKBIA*, 5′-TGAAGGCTACCAACTACAATGGC-3′ (forward), and 5′-TGACATCAGCACCCAAGGACAC-3′ (reverse). The expression of cognate mRNA was normalized by the geometric mean of the housekeeping genes *HPRT1*, 5′-TGACACTGGCAAAACAATGCA-3′ (forward), and 5′-GGTCCTTTTCACCAGCAAGCT-3′ (reverse); *HMBS*, 5′-GGCAATGCGGCTGCAA-3′ (forward), and 5′-GGGTACCCACGCGAATCAC-3′ (reverse); and *RPL13A*, 5′-CCTGGAGGAGAAGAGGAAAGAGA-3′ (forward), and 5′-TTGAGGACCTCTGTGTATTTGTCAA-3′ (reverse), as previously described [29].

### 2.4. Analysis of Interleukin Production

Cell culture supernatants from monocytes treated with 1 μg/mL N and S SARS-CoV-2 proteins for 3 days were analyzed by a Human Inflammation Array C3 (AAH-INF-3) from RayBiotech (Atlanta, GA, USA). Chemiluminescence was detected using the G-Box chemiluminescence imaging station (Syngene, Cambridge, UK). Quantification was performed using Analysis Tool for AAH-INF-3 from RayBiotech (Atlanta, GA, USA). IL-6 production in the monocyte and macrophage cellular supernatants was analyzed using a Human IL-6 Quantikine ELISA Kit (R&D Systems), according to the manufacturer’s instructions.

### 2.5. Statistics

The results were analyzed using Friedman repeated measures ANOVA on ranks followed by Student–Newman–Keuls post hoc test, and the analysis was performed using SigmaStat ver. 3.5 (Systat Software Inc., San Jose, CA, USA). Differences were considered statistically significant at *p* ˂ 0.05. 

## 3. Results

**Figure 1:**
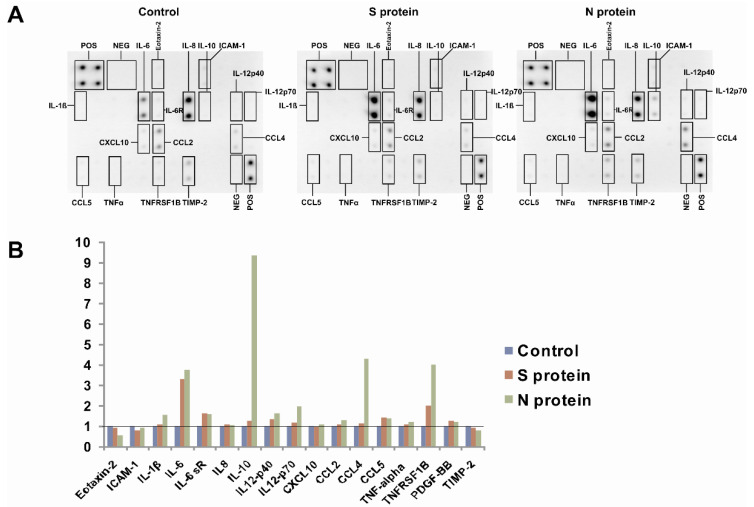
Effect of the S and N proteins of the severe acute respiratory syndrome coronavirus 2 (SARS-CoV-2) virus on the induction of inflammatory proteins in monocytes. (**A**) Human monocytes isolated from peripheral blood mononuclear cells (PBMCs) were cultured for 3 days in the presence of 1 μg/mL S protein and 1 μg/mL N protein. Control cells were treated with an equal volume of phosphate-buffered saline (PBS). The supernatants from these cultures were analyzed by the Human Inflammation Array C3 (AAH-INF-3, RayBiotech). (**B**) Quantification of dots from upper panel performed using Analysis Tool for AAH-INF-3 (RayBiotech). Results are shown as fold induction over control-treated cells.

**Figure 2:**
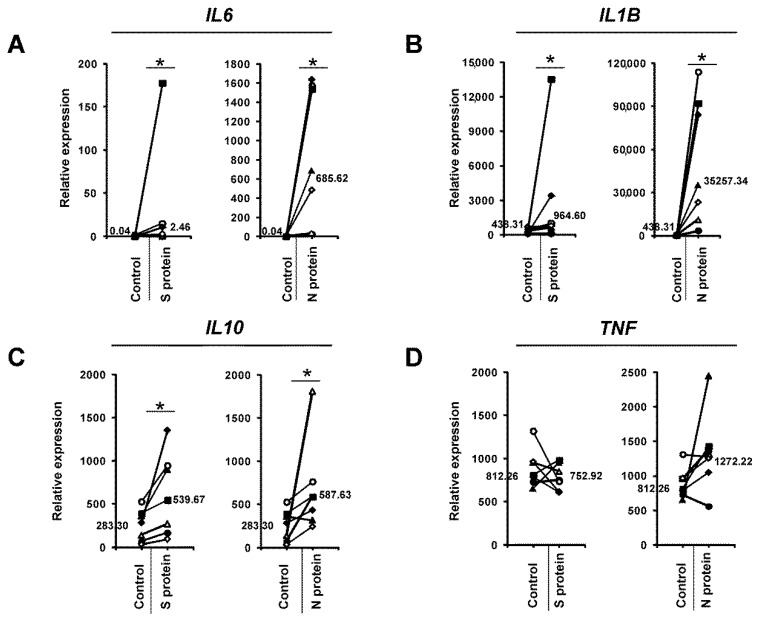
mRNA expression of the *IL6* (**A**), *IL1B* (**B**), *IL10* (**C**), and *TNF* (**D**) genes in monocytes cultured in the presence of 1 μg/mL SARS-CoV-2 proteins S (spike) and N (nucleocapsid) for 3 days. Control cells were treated with an equal volume of PBS. The expression of each mRNA was determined by real-time RT-PCR and normalized to the mRNA levels of the housekeeping genes *HPRT1*, *HMBS*, and *RPL13A*, as described in the Materials and Methods section. The data show individual values for seven independent donors (*n* = 7), and straight lines connect the control value and the value after SARS-CoV-2 protein treatment from individual donors. The median values are also shown in the figures. An asterisk indicates a statistically significant difference at *p* < 0.05.

**Figure 3:**
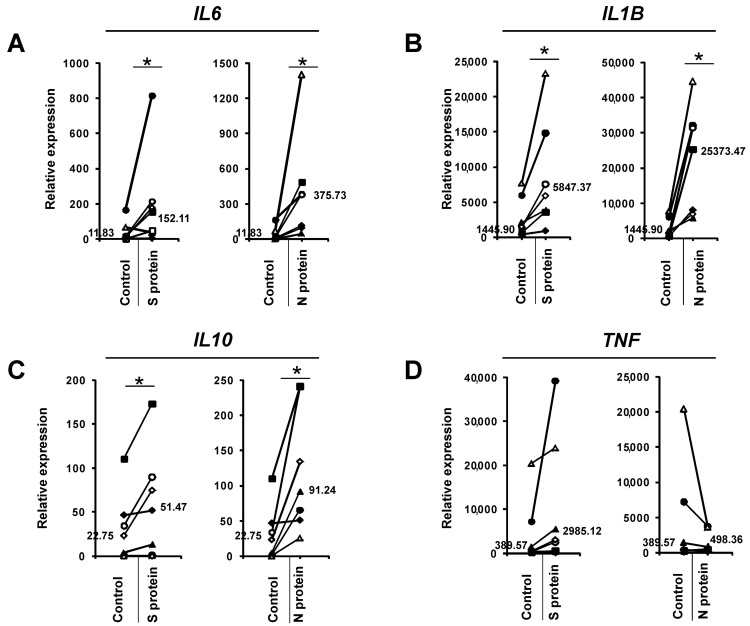
mRNA expression of *IL6* (**A**), *IL1B* (**B**), *IL10* (**C**), and *TNF* (D) genes in monocytes differentiated towards macrophages in the presence of 1 μg/mL SARS-CoV-2 proteins S (spike) and N (nucleocapsid) for 5 days. Control cells were treated with an equal volume of PBS. The expression of each mRNA was determined by real-time RT-PCR and normalized to the mRNA levels of the housekeeping genes *HPRT1*, *HMBS*, and *RPL13A*, as described in the Materials and Methods section. The data show individual values for seven independent donors (*n* = 7), and straight lines connect the control value and the value after SARS-CoV-2 protein treatment from individual donors. The median values are also shown in the figures. An asterisk indicates a statistically significant difference at *p* < 0.05.

**Figure 4:**
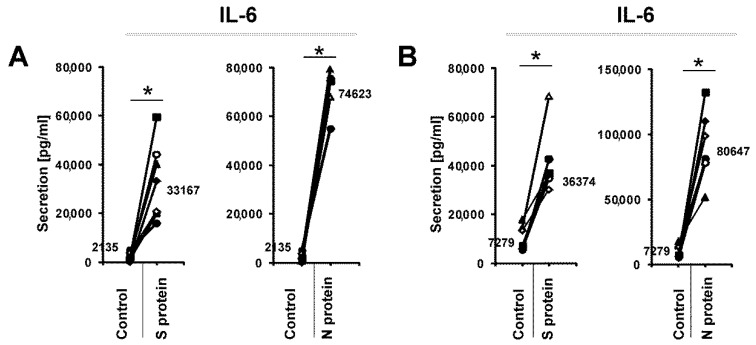
IL-6 production in the cellular supernatants of human monocytes (**A**) and macrophages (**B**) cultured in the presence of 1 μg/mL SARS-CoV-2 proteins S (spike) and N (nucleocapsid) was determined using the human IL-6 Quantikine ELISA Kit. Control cells were treated with an equal volume of PBS. The data show individual values for seven independent donors (*n* = 7), and straight lines connect the control value and the value after SARS-CoV-2 protein treatment from individual donors. The median values are also shown in the figures. An asterisk indicates a statistically significant difference at *p* < 0.05.

We initially wanted to determine if SARS-CoV-2 proteins might change the profile of cytokines released from human monocytes. To this end, we cultured human monocytes for 3 days in the presence of 1 μg/mL S (spike) and N (nucleocapsid) SARS-CoV-2 proteins and then analyzed the supernatants using a cytokine array. As shown in Figure 1, the N protein caused induction of IL-6, IL-6R, IL-1β, IL-10, IL-12, CCL4, and TNFRSF1B compared to the control treatment. Interestingly, the effect of the S protein was lower (Figure 1). Next, we confirmed this initial observation by analyzing monocytes from a larger number of donors to confirm if the N and S proteins induced the expression of *IL6* and other cytokine genes at the mRNA level. This more detailed analysis confirmed our initial results: the N protein of the SARS-CoV-2 coronavirus led to strong induction of *IL6* mRNA expression, while the S protein was much less effective (Figure 2A). The observed effect was dose-dependent and specific for active proteins, while boiled S and N proteins and BSA (bovine serum albumin) were ineffective in inducing *IL6* in monocytes (Table S1). Similarly, the expression of the proinflammatory cytokine *IL1B* was also substantially increased in the monocytes treated with the N protein and, to a lesser extent, with the S protein (Figure 2B). While *IL10* expression was also induced, there were no significant differences between the N protein- and S protein-treated cells (Figure 2C). Interestingly, we did not observe the effects of these viral proteins on *TNF* expression (Figure 2D). Next, we analyzed the effects of the SARS-CoV-2 proteins in macrophages differentiated from monocytes from the same donors. As seen in Figure 3A, the SARS-CoV-2 proteins caused an upregulation of *IL6* expression in the monocyte-derived macrophages, but to a lesser extent than in the monocytes, with a smaller difference between the effects of N and S proteins. This result might be explained by the different receptor repertoires and signaling pathways in monocytes and macrophages that may lead to changes in the response of these cells to different stimuli [30]. The expression patterns of *IL1B*, *IL10*, and *TNF* in the macrophages treated with the SARS-CoV-2 proteins were similar to those in the monocytes treated with the SARS-CoV-2 proteins (Figure 3B–D). Increased secretion of the IL-6 protein by the monocytes and macrophages cultured in the presence of the N and S SARS-CoV-2 proteins was observed by ELISA, and this analysis confirmed that the N protein was a stronger inducer of IL-6 than the S protein (Figure 4). Previous studies have shown that SARS-CoV-1 proteins induce IL-6 expression in monocytes/macrophages and dendritic cells in an NF-κB-dependent manner [31,32,33]. Recent work by Patra et al. indicated that the spike protein of SARS-CoV-2 induces expression of IL-6 and IL-6R in epithelial cells via activation of NF-κB [34]. This prompted us to investigate if the S and N proteins of SARS-CoV-2 could influence the transcription of genes encoding some elements of the NF-κB signaling pathway. Results showed that monocytes exposed to S and N proteins of SARS-CoV-2 express higher levels of *NFKB1* (Nuclear Factor Kappa B Subunit 1, p50) and *NFKBIA* (NFKB Inhibitor Alpha, IκBα) mRNA, whilst *RELA* (RELA Proto-Oncogene, NF-KB Subunit, p65) was unaffected (Figure S1). Interestingly, in macrophages exposed to S and N proteins, we observed significant induction of only the *NFKBIA* mRNA (Figure S2). 

## 4. Discussion

Despite the substantial progression of knowledge about SARS-CoV-2 and the disease it causes, we still do not have a complete understanding of the immune response to this virus. Monocytes and macrophages, due to their cytokine production, phagocytosis, presentation of antigens, and interaction with other cells, play a critical role in effective antiviral immune responses [35]. However, monocytes and macrophages can also be involved in virus persistence and dissemination [18], and their hyperactivation can lead to immunopathological reactions [35,36,37]. Here, we show, for the first time, that monocytes and macrophages cultured in the presence of the S and N proteins of the novel SARS-CoV-2 coronavirus express high levels of *IL6* and other cytokines, such as *IL1B* and *IL10*. This is similar to the previously observed effect of SARS-CoV-1 proteins [19,31,32,33]. However, the pattern of SARS-CoV-1 S protein-mediated cytokine expression in monocytes was different than the pattern observed in this study. While the SARS-CoV-1 S protein upregulated IL-6, IL-8, and TNF, the SARS-CoV-2 S protein upregulated IL-6 but not IL-8 or TNF (Figure 1 and Figure 2). Analysis of the expression of *RELA*, *NFKB1*, and *NFKBIA* (Figure S1) in cells exposed to S and N SARS-CoV-2 proteins indicated that *NFKB1* and *NFKBIA* were induced in monocytes and *NFKBIA* was induced in macrophages (Figures S1 and S2). High mRNA expression of *NFKBIA* is commonly associated with the activation of NF-κB signaling [38,39,40], therefore NF-κB might be involved in the observed process of IL-6 induction by SARS-CoV-2 proteins, similarly to previous reports on SARS-CoV-1 proteins-mediated IL-6 induction [31,32,33]. However, it should be noted that we have no direct evidence that this is the case and further research is required. Interestingly, the SARS-CoV-2 S protein also showed an activating effect on cytokine expression in Th1 cells [27]. However, in contrast to monocytes, which showed a higher response to the SARS-CoV-2 N protein (Figure 2A), Th1 cells did not show such a preference [27], which is somewhat contradictory to the results of a bioinformatics study by Dhall et al. [41]. This observation might be of importance in the context of developing vaccines based on SARS-CoV-2 proteins, as it suggests that SARS-CoV-2 N protein-based vaccines might be more likely to induce a Th2 immune response, leading to humoral immunity, than vaccines utilizing the SARS-CoV-2 S protein. Interestingly, we also observed substantial differences in the response to the N protein between donors. The level of *IL6* mRNA induction varied between 30-fold and several thousand-fold (Figure 2A and DataSet S1). This finding is consistent with the hypothesis that genetic diversity (polymorphisms, mutations) in proteins involved in recognizing viral proteins, such as pattern recognition receptors and signaling molecules, may lead to different risks of more intense and unbalanced inflammatory responses and subsequent cytokine storm syndrome.

**Figure 5:**
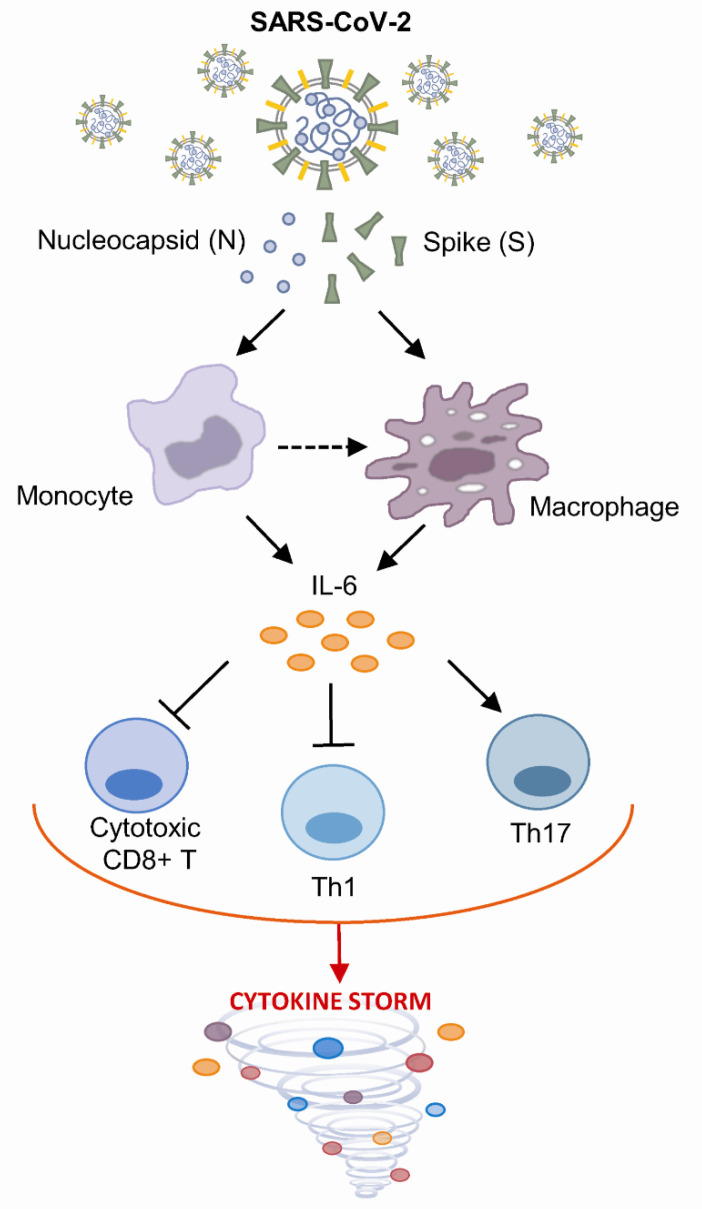
Schematic presentation of SARS-CoV-2 protein-dependent activation of monocytes/macrophages, leading to IL-6 release and cytokine storm.

Several studies have suggested that cytokine storms and acute respiratory distress syndrome are associated with high levels of proinflammatory cytokines, including very high levels of IL-6, that correlate with the severity of disease [42,43], especially in patients with a high plasma viral load [44]. Furthermore, COVID-19 patients have a larger number of activated monocytes that infiltrate the lungs and lead to damage [45]. IL-6 is secreted by monocytes and macrophages and might play a central role in SARS-CoV-2-induced cytokine storms [46]; this cytokine inhibits the Th1 response [47], which is observed in severe COVID-19 patients [48], and it attenuates cytotoxic CD8+ T cell activity [49] and promotes differentiation and Th17-dependent virus persistence and immunopathology (Figure 5) [50].

## 5. Conclusions

Tocilizumab, an immunosuppressive drug blocking IL-6 receptors, has shown a seemingly positive effect in patients with COVID-19 [51,52]; however, because of adverse effects, new candidate molecules that are more effective and/or act on the level of transcription or translation of IL-6 are needed [23]. An alternative option is to use agents that interfere with monocyte infiltration or affect the signaling pathways that lead to SARS-CoV-2 protein-dependent IL-6 induction in monocytes. Such a strategy has already shown promising results in clinical studies of the Bruton tyrosine kinase (BTK) inhibitor acalabrutinib. BTK is a known regulator of macrophage activation, and treatment of COVID-19 patients with acalabrutinib has been shown to improve oxygenation as well as normalization of IL-6 levels [53]. Determining the molecular mechanism of SARS-CoV-2-mediated monocyte and macrophage IL-6 expression, including identifying the receptors and signaling pathways, would allow for the rational design of pharmacological treatments for the cytokine storm that underlies the severe clinical manifestation of COVID-19 disease. Such investigations are now in progress in our laboratory.

## Data Availability

All data are available on request. Please contact: mratajewski@cbm.pan.pl.

## Supplementary Materials

The following are available online at https://www.mdpi.com/2076-393X/9/1/54/s1: Table S1: mRNA expression of *IL6* gene in monocytes cultured in the presence of increasing concentrations of SARS-CoV-2 proteins S (spike) and N (nucleocapsid), 1 μg/mL of the boiled (1 h, 99 °C) SARS-CoV-2 proteins S (spike) and N (nucleocapsid) and 1 μg/mL of BSA for 3 days; Figure S1: mRNA expression of *RELA*, *NFKB1*, and *NFKBIA* genes in monocytes cultured in the presence of 1 μg/mL SARS-CoV-2 proteins S (spike) and N (nucleocapsid) for 3 days; Figure S2: mRNA expression of *RELA*, *NFKB1*, and *NFKBIA* genes in monocytes differentiated towards macrophages in the presence of 1 μg/mL SARS-CoV-2 proteins S (spike) and N (nucleocapsid) for 5 days; DataSet S1: datasets for Figure 2, Figure 3 and Figure 4, Figures S1 and S2.
