# Supplementary material for: Nucleocapsid and Spike Proteins of the Coronavirus SARS-CoV-2 Induce IL6 in Monocytes and Macrophages—Potential Implications for Cytokine Storm Syndrome
Source: Vaccines (Basel). 2021 Jan 15;9(1):54. doi: 10.3390/vaccines9010054 (PMC7830532; doi:10.3390/vaccines9010054)
Supplement: Supplementary file 1 [file vaccines-09-00054-s001.zip › vaccines-1061897-xml-sup/vaccines-1061897-xml-sup.pdf]

Supporting information

Nucleocapsid and spike proteins of the coronavirus SARS-CoV-2 induce IL6 in monocytes and macrophages – potential implications for cytokine storm syndrome.

Iwona Karwaciak, Anna Sałkowska, Kaja Karaś, Jarosław Dastych and Marcin Ratajewski

Table of contents

1. Table S1……………………………………………………S3
2. Figure S1……………………………………………………S4
3. Figure S2……………………………………………………S5

Donor	Control	Protein S [0.1 µg/ml]	Protein S [0.4 µg/ml]	Protein S [1 µg/ml]	Boiled protein S [1 µg/ml]	BSA [1 µg/ml]
8	**0.72**	**0.63**	**2.66**	**5.76**	**0.71**	**1.21**

Donor	Control	Protein N [0.1 µg/ml]	Protein N [0.4 µg/ml]	Protein N [1 µg/ml]	Boiled protein N [1 µg/ml]	BSA [1 µg/ml]
9	**1.34**	**57.65**	**876.50**	**1870.21**	**0.96**	**1.50**

Table S1. mRNA expression of *IL6* gene in monocytes cultured in the presence of increasing concentrations SARS-CoV-2 proteins S (spike) and N (nucleocapsid), 1 µg/ml of the boiled (1h, 99°C) SARS-CoV-2 proteins S (spike) and N (nucleocapsid) and 1 µg/ml of BSA for 3 days. Control cells were treated with the equal volume of PBS. The expression of each mRNA was determined by real-time RT-PCR and normalized to the mRNA levels of the housekeeping genes *HPRT1*, *HMBS* and *RPL13A*, as described in the Materials and Methods.

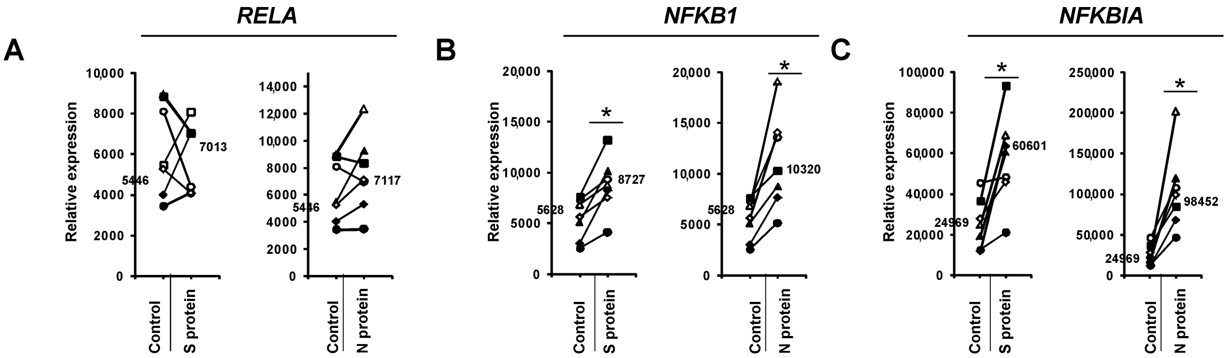

Figure S1. mRNA expression of *RELA* (A), *NFKB1* (B) and *NFKBIA* (C) genes in monocytes cultured in the presence of 1 µg/ml SARS-CoV-2 proteins S (spike) and N (nucleocapsid) for 3 days. Control cells were treated with an equal volume of PBS. The expression of each mRNA was determined by real-time RT-PCR and normalized to the mRNA levels of the housekeeping genes *HPRT1*, *HMBS* and *RPL13A*, as described in the Materials and Methods. The data show individual values for seven independent donors (n = 7), and straight lines connect the control value and the value after SARS-CoV-2 protein treatment from individual donors. The median values are also shown in the figures. An asterisk indicates a statistically significant difference at P < 0.05.

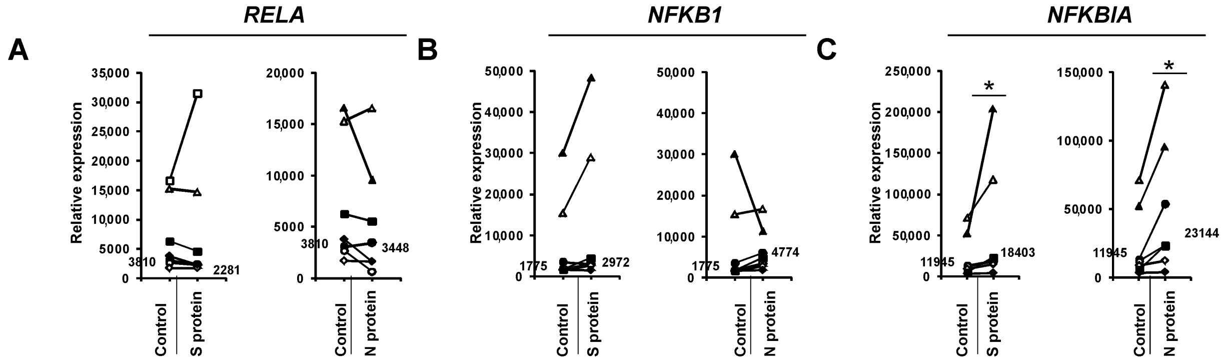

Figure S2. mRNA expression of *RELA* (A), *NFKB1* (B) and *NFKBIA* (C) in monocytes differentiated towards macrophages in the presence of 1 µg/ml SARS-CoV-2 proteins S (spike) and N (nucleocapsid) for 5 days. Control cells were treated with an equal volume of PBS. The expression of each mRNA was determined by real-time RT-PCR and normalized to the mRNA levels of the housekeeping genes *HPRT1*, *HMBS* and *RPL13A*, as described in the Materials and Methods. The data show individual values for seven independent donors (n = 7), and straight lines connect the control value and the value after SARS-CoV-2 protein treatment from individual donors. The median values are also shown in the figures. An asterisk indicates a statistically significant difference at P < 0.05.
