# Supplementary material for: Nucleocapsid and Spike Proteins of the Coronavirus SARS-CoV-2 Induce IL6 in Monocytes and Macrophages—Potential Implications for Cytokine Storm Syndrome
Source: Vaccines (Basel). 2021 Jan 15;9(1):54. doi: 10.3390/vaccines9010054 (PMC7830532; doi:10.3390/vaccines9010054)
Supplement: Supplementary file 1 [file vaccines-09-00054-s001.zip › vaccines-1061897-xml-sup/Supplementary information_revision.doc]

Supporting information

Nucleocapsid and spike proteins of the coronavirus SARS-CoV-2 induce IL6 in monocytes and macrophages – potential implications for cytokine storm syndrome.

Iwona Karwaciak, Anna Sałkowska, Kaja Karaś, Jarosław Dastych and Marcin Ratajewski

Table of contents

1. Table S1…………………………………………………………S3
2. Figure S1…………………………………………………………S4
3. Figure S2…………………………………………………………S5

| Donor | Control | Protein S [0.1 μg/ml] | Protein S [0.4 μg/ml] | Protein S [1 μg/ml] | Boiled protein S [1 μg/ml] | BSA [1 μg/ml] |
| --- | --- | --- | --- | --- | --- | --- |
| 8 | **0.72** | **0.63** | **2.66** | **5.76** | **0.71** | **1.21** |

| Donor | Control | Protein N [0.1 μg/ml] | Protein N [0.4 μg/ml] | Protein N [1 μg/ml] | Boiled protein N [1 μg/ml] | BSA [1 μg/ml] |
| --- | --- | --- | --- | --- | --- | --- |
| 9 | **1.34** | **57.65** | **876.50** | **1870.21** | **0.96** | **1.50** |

**Table S1.** mRNA expression of *IL6* gene in monocytes cultured in the presence of increasing concentrations SARS-CoV-2 proteins S (spike) and N (nucleocapsid), 1 μg/ml of the boiled (1h, 99^o^C) SARS-CoV-2 proteins S (spike) and N (nucleocapsid) and 1 μg/ml of BSA for 3 days. Control cells were treated with the equal volume of PBS. The expression of each mRNA was determined by real-time RT-PCR and normalized to the mRNA levels of the housekeeping genes *HPRT1*, *HMBS* and *RPL13A*, as described in the Materials and Methods.

**Figure S1.** mRNA expression of *RELA* (A), *NFKB1* (B) and *NFKBIA* (C) genes in monocytes cultured in the presence of 1 μg/ml SARS-CoV-2 proteins S (spike) and N (nucleocapsid) for 3 days. Control cells were treated with an equal volume of PBS. The expression of each mRNA was determined by real-time RT-PCR and normalized to the mRNA levels of the housekeeping genes *HPRT1*, *HMBS* and *RPL13A*, as described in the Materials and Methods. The data show individual values for seven independent donors (n = 7), and straight lines connect the control value and the value after SARS-CoV-2 protein treatment from individual donors. The median values are also shown in the figures. An asterisk indicates a statistically significant difference at P < 0.05.

**Figure S2.** mRNA expression of *RELA* (A), *NFKB1* (B) and *NFKBIA* (C) in monocytes differentiated towards macrophages in the presence of 1 μg/ml SARS-CoV-2 proteins S (spike) and N (nucleocapsid) for 5 days. Control cells were treated with an equal volume of PBS. The expression of each mRNA was determined by real-time RT-PCR and normalized to the mRNA levels of the housekeeping genes *HPRT1*, *HMBS* and *RPL13A*, as described in the Materials and Methods. The data show individual values for seven independent donors (n = 7), and straight lines connect the control value and the value after SARS-CoV-2 protein treatment from individual donors. The median values are also shown in the figures. An asterisk indicates a statistically significant difference at P < 0.05.
